# Gamma knife radiosurgery for patients with brain metastases from non–small cell lung cancer: Comparison of survival between <5 and ≥5 metastases

**DOI:** 10.1111/1759-7714.14532

**Published:** 2022-06-29

**Authors:** Xu Zhao, Shouluan Ding, Ming Zhang, Chengwei Wang

**Affiliations:** ^1^ Department of Neurosurgery The Second Hospital of Shandong University Jinan China; ^2^ Institute of Medical Sciences The Second Hospital of Shandong University Jinan China

**Keywords:** brain metastases, gamma knife, non–small cell lung cancer, stereotactic radiosurgery

## Abstract

**Background:**

Current evidence‐based guidelines support stereotactic radiosurgery (SRS) for patients with up to four brain metastases (BMs). However, debate continues about how many tumors may be treated by SRS alone.

**Methods:**

This retrospective study included non–small cell lung cancer (NSCLC) patients with BMs treated with gamma knife as the initial treatment for cerebral lesions. The patients were followed up to obtain their survival information. The outcomes were statistically analyzed to compare the differences in survival between the <5 BMs and ≥5 BMs groups and to identify prognostic factors.

**Results:**

A total of 77 patients were divided into two groups (54 patients with <5 BMs and 23 patients with ≥5 BMs). The median overall survival (OS) was 18.3 months in the <5 BMs group and 17.7 months in the ≥5 BMs group. The median intracranial progression‐free survival (IPFS) was 9.0 months in the <5 BMs group and 9.9 months in the ≥5 BMs group. There was no significant difference in OS and IPFS between the two groups. The multivariate analysis demonstrated that adenocarcinoma, controlled primary cancer, higher Karnofsky Performance Scale (KPS), and salvage treatment were independent prognostic factors favoring longer OS.

**Conclusion:**

SRS alone as the initial treatment for NSCLC patients with more than four BMs was non‐inferior to SRS for those with one to four BMs in terms of OS and IPFS.

## Graphical abstract

SRS alone as the initial treatment for NSCLC patients with more than four BMs was non‐inferior to SRS for those with one to four BMs in terms of OS and IPFS. Adenocarcinoma, controlled primary cancer, higher KPS, and salvage treatment were independent prognostic factors favoring longer OS.

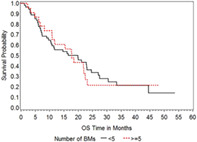

## INTRODUCTION

Brain metastases (BMs) are a critical issue for patients with cancer, because they can significantly negatively affect patients' quality of life and survival. Up to 40% of the patients diagnosed with non–small cell lung cancer (NSCLC) would develop BMs during the disease.^1^, ^2^ Treatment options for BMs include surgery, whole brain radiation therapy (WBRT), stereotactic radiosurgery (SRS), chemotherapy, and a combination of these modalities. The standard of care for patients with BMs has not been clearly defined, and the practice patterns vary among oncologists, radiation oncologists, and neurosurgeons.^3^, ^4^, ^5^

SRS has been used for decades in the treatment of BMs. Several early studies demonstrated that patients with ≥5 BMs had worse overall survival (OS) after receiving SRS than patients with 1–4 BMs.^6^, ^7^, ^8^, ^9^ Therefore, WBRT is recommended to treat patients with ≥5 BMs. This assumption, however, has increasingly been challenged in the current era of improved systemic therapy and survival.^10^, ^11^ Recent reports showed a trend to treat patients with ≥5 BMs by SRS alone.^12^, ^13^, ^14^

Based on clinical data of NSCLC patients with BMs treated with SRS, we conducted this retrospective study to investigate whether patients with ≥5 BMs had worse survival than patients with 1–4 BMs.

## METHODS

### Patient population

All NSCLC patients with BMs initially treated with gamma knife at the Second Hospital of Shandong University (China) from February 2016 to January 2021 were candidates for this retrospective study. Patients were included in this study if they met the following inclusion criteria. First, they had pathologically confirmed NSCLC and newly diagnosed BMs. Second, they had no history of prior treatment with SRS, WBRT, or surgery for BMs. Third, the maximum diameter of the largest BMs was ≤5 cm. Fourth, there was no apparent leptomeningeal dissemination. The institutional review board of our hospital approved the use of patient records for this clinical research.

### Gamma knife radiosurgery techniques

SRS was performed using the Gamma Knife Perfexion (Elekta AB). After conscious sedation and local anesthesia, a stereotactic head frame (Leksell Model G) was attached. The stereotactic gadolinium‐enhanced T_1_‐weighted axial magnetic resonance (MR) images with a slice thickness of 2 mm were obtained for target coordinate determination and dose planning. SRS treatment was planned with GammaPlan software 10.1.1 (Elekta AB). All BMs were treated at a margin dose of 12–24 Gy with an isodose line of 40%–80%. Dose selection depended on tumor volume and proximity to critical structures such as the brainstem and optic nerve. In general, smaller tumors distant from critical structures were treated with higher margin doses (20–24 Gy); larger tumors close to vital structures received lower margin doses (12–18 Gy).

### Follow‐up

Clinical and imaging follow‐ups were usually performed 3 months after SRS, then every 3 to 6 months, or sooner (if indicated). Survival time was obtained from medical records or telephone follow‐up. OS was defined as the interval between SRS and death from any cause, and the outcome was censored if a patient was alive at the last follow‐up. Intracranial progression‐free survival (IPFS) was defined as the interval from SRS to the detection of any intracranial disease progression or death. The outcome was censored if a patient was alive without known intracranial disease progression at the last follow‐up. Repeat SRS or subsequent WBRT was recommended if tumor growth or new metastases were identified. Radionecrosis was diagnosed if patients had magnetic resonance imaging (MRI) changes consistent with necrosis in the setting of new neurologic symptoms or a new steroid requirement. In asymptomatic patients, the diagnosis of radionecrosis was based on MRI (including perfusion‐weighted imaging and MR spectroscopy) and positron emission computed tomography (PET).

### Statistical analysis

Differences in OS and IPFS were compared between patients with <5 BMs and those with ≥5 BMs, and prognostic factors related to OS and IPFS were identified. We used the *t*‐test or Wilcoxon rank‐sum test for continuous variables and the χ^2^ test or Fisher's exact test for categorical variables to detect the differences in patient characteristics between the two groups. We used the Mantel–Haenszel χ^2^ test to calculate the correlation between the number of BMs, primary cancer status, and extracranial metastases. Estimated survival was calculated according to the Kaplan–Meier method. The difference in survival curves between the groups was assessed using the log‐rank test. Univariate Cox regression followed by multivariable Cox proportional hazards regression was used to calculate hazard ratios (HRs) and identify independent prognostic factors for survival. All statistical analyses were performed using R software version4.1.3. A *p*‐value <0.05 was considered statistically significant.

## RESULTS

### Patient characteristics

The follow‐up duration from SRS ranged from 2 to 60 months. The detailed patient characteristics are summarized in Table 1. Seventy‐seven NSCLC patients with BMs were divided into two groups according to the number of metastases; 54 patients had <5 BMs, and 23 had ≥5 BMs. There were significant differences in extracranial metastases, primary cancer status, and total volume of BMs between the two groups. Compared with the <5 BMs group, the ≥5 BMs group included more patients with extracranial metastases and uncontrolled primary cancer. The mean total volume of BMs was larger in the ≥5 BMs group than that in the <5 BMs group. About salvage treatment for BMs, 17 of 19 patients in the <5 BMs group received repeat SRS, and the remaining two patients received WBRT. In contrast, all eight patients in the ≥5 BMs group received repeat SRS.

**TABLE 1:** Patient characteristics

Characteristics	Total (*n* = 77)	<5 group (*n* = 54)	≥5 group (*n* = 23)	*P* value
Age in y, median (range)	60.8 (30–82)	60.1 (41–82)	62.3 (30–79)	0.4424
Sex				
Male	44 (57.1%)	32 (59.3%)	12 (52.2%)	0.5653
Female	33 (42.9%)	22 (40.7%)	11 (47.8%)	
Histology				
Adenocarcinoma	58 (75.3%)	40 (74.1%)	18 (78.3%)	1.0000
Squamous carcinoma	14 (18.2%)	10 (18.5%)	4 (17.4%)	
Others	5 (6.5%)	4 (7.4%)	1 (4.3%)	
Symptoms from BMs				
Yes	46 (59.7%)	32 (59.3%)	14 (60.9%)	0.8951
No	31 (40.3%)	22 (40.7%)	9 (39.1%)	
Extracranial metastases				
Present	21 (27.3%)	10 (18.5%)	11 (47.8%)	0.0082
Absent	56 (72.7%)	44 (81.5%)	12 (52.2%)	
Primary cancer status				
Controlled	35 (45.5%)	30 (55.6%)	5 (21.7%)	0.0064
Not controlled	42 (54.5%)	24 (44.4%)	18 (78.3%)	
Number of BMs, mean (range)	3.7 (1–21)	1.9 (1–4)	7.9 (5–21)	–
Maximum diameter of BMs in cm, mean (range)	2.3 (0.7–4.9)	2.2 (0.7–4.9)	2.4 (0.9–4.5)	0.4065
Total volume of BMs in cm^3^, mean (range)	12.7 (0.5–99.4)	7.7 (0.5–48.9)	24.5 (1.8–99.4)	0.0001
KPS, mean (range)	81.4 (30–100)	80.6 (30–100)	83.5 (50–100)	0.3667
Prior chemotherapy				
Yes	50 (64.9%)	38 (70.4%)	12 (52.2%)	0.1256
No	27 (35.1%)	16 (29.6%)	11 (47.8%)	
Prior targeted therapy				
Yes	27 (35.1%)	21 (38.9%)	6 (26.1%)	0.2813
No	50 (64.9%)	33 (61.1%)	17 (73.9%)	
Salvage treatment				
Yes	27 (35.1%)	19 (35.2%)	8 (34.8%)	0.9730
No	50 (64.9%)	35 (64.8%)	15 (65.2%)	

Abbreviations: BMs, brain metastases; KPS, Karnofsky Performance Scale; y, years.

In addition, Spearman's rank correlation analysis showed significant correlations among extracranial metastases, number of BMs, and primary cancer status. Specifically, extracranial metastases versus number of BMs (r_s_ = 0.30, *p* = 0.0086), primary cancer status versus number of BMs (r_s_ = −0.31, *p* = 0.0067), and extracranial metastases versus primary cancer status (r_s_ = −0.27, *p* = 0.0203).

### Survival data in the <5 BMs group and ≥5 BMs group

**FIGURE 1:**
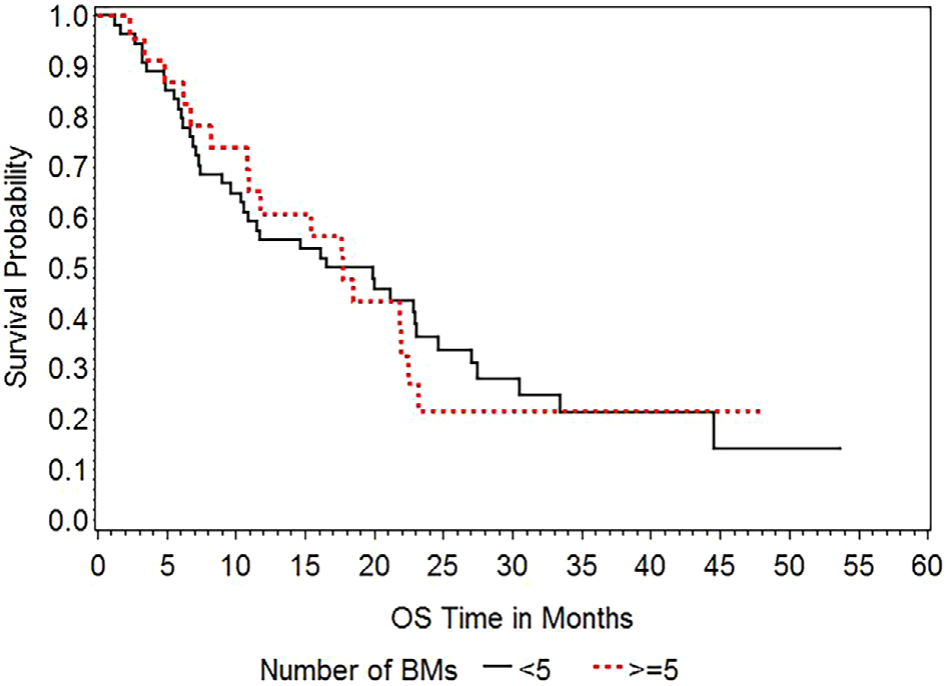
Kaplan–Meier curves of overall survival according to the number of BMs (<5 BMs group vs. ≥5 BMs group)

At last follow‐up, 56 patients had died, including 40 with lung cancer‐related systemic progression, seven with intracranial progression, one with noncancerous causes, and eight with indeterminate causes. The median OS for all patients was 17.7 (95% confidence interval [CI], 10.9–22.4) months. The cumulative post‐SRS OS rates were 83.1% at 6 months, 57.1% at 1 year, and 32.3% at 2 years. Figure 1 shows the Kaplan–Meier curves of OS of the <5 BMs and ≥5 BMs groups. The median OS was 18.3 (95% CI, 10.4–23.1) months in the <5 BMs group and 17.7 (95% CI, 10.8–22.4) months in the ≥5 BMs group. There was no significant difference in OS between the two groups (*p* = 0.8553).

**FIGURE 2:**
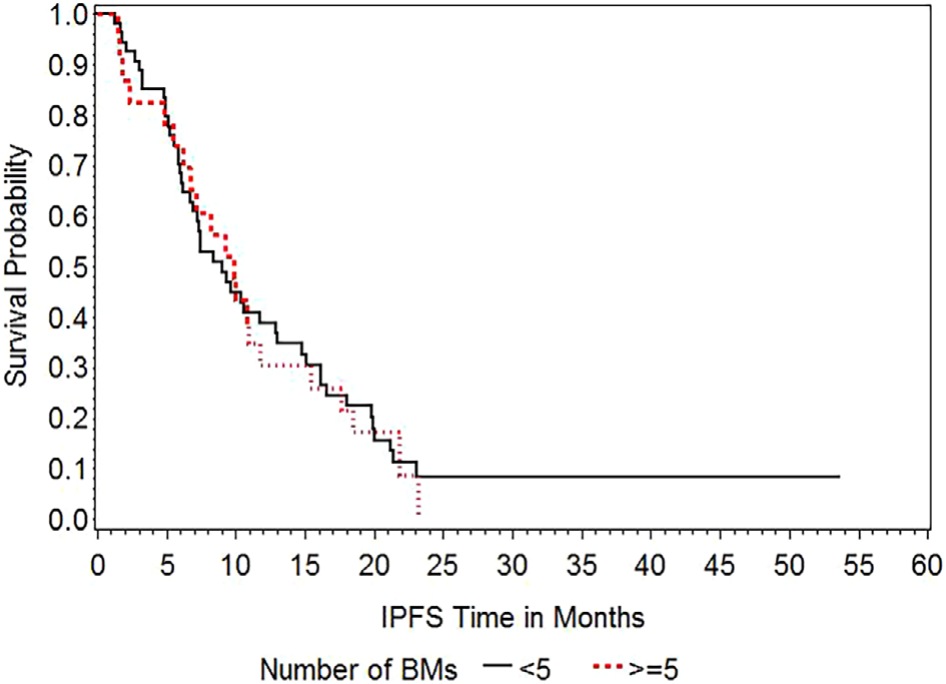
Kaplan–Meier curves of intracranial progression‐free survival according to the number of BMs (<5 BMs group vs. ≥5 BMs group)

Local tumor recurrence was developed in 5 (6.5%) patients, whereas new brain metastases at other sites occurred in 48 (62.3%) patients. Figure 2 shows the Kaplan–Meier curves of IPFS of the <5 BMs and ≥5 BMs groups. The median IPFS for all patients from SRS was 9.2 (95% CI, 7.1–10.9) months. The median IPFS was 9.0 (95% CI, 6.7–12.9) months in the <5 BMs group and 9.9 (95% CI, 6.2–11.7) months in the ≥5 BMs group. There was no significant difference in IPFS between the two groups (*p* = 0.8384).

### Prognostic factors for OS and IPFS

Prognostic factors for OS identified by univariate and multivariate analyses are shown in Table 2. The multivariate analysis demonstrated that adenocarcinoma, controlled primary cancer, higher KPS, and salvage treatment were significant prognostic factors favoring more prolonged survival. We also performed univariate and multivariate analyses to identify prognostic factors for IPFS, which are summarized in Table 3. Age, histology, primary cancer status, prior chemotherapy, and salvage treatment were statistically significant prognostic factors for IPFS.

**TABLE 2:** Univariate and multivariate analyses of covariates associated with overall survival

Variables	Univariate analysis			Multivariate analysis		
	HR	95% CI	*P* value	HR	95% CI	*P* value
Age, y	1.00	0.97–1.02	0.8677			
Sex (male vs. female)	1.56	0.91–2.69	0.1047			
Histology (non‐Ad vs. Ad)	3.15	1.72–5.76	0.0002	3.01	1.56–5.78	0.0010
Symptoms from BMs (yes vs. no)	1.61	0.93–2.81	0.0920			
Extracranial metastases (present vs. absent)	1.31	0.73–2.34	0.3675			
Primary cancer status (controlled vs. not controlled)	0.68	0.40–1.16	0.1529	0.51	0.29–0.90	0.0204
Number of BMs	1.00	0.93–1.07	0.9025			
Maximum diameter	1.08	0.87–1.35	0.4763			
Total volume of BMs	1.00	0.98–1.01	0.8619			
KPS	0.98	0.96–0.99	0.0064	0.98	0.96–1.00	0.0124
Prior chemotherapy (yes vs. no)	1.58	0.90–2.79	0.1125			
Prior targeted therapy (yes vs. no)	0.55	0.31–0.98	0.0436			
Salvage treatment (yes vs. no)	0.48	0.27–0.86	0.0130	0.52	0.28–0.97	0.0403

Abbreviations: Ad, adenocarcinoma; BMs, brain metastases; CI, confidence interval; HR, hazard ratio; KPS, Karnofsky Performance Scale; y, years.

**TABLE 3:** Univariate and multivariate analyses of covariates associated with intracranial progression‐free survival

Variables	Univariate analysis			Multivariate analysis		
	HR	95% CI	*P* value	HR	95% CI	*P* value
Age, y	1.01	0.99–1.04	0.1907	1.03	1.01–1.06	0.0174
Sex (male vs. female)	1.18	0.73–1.93	0.4982			
Histology (non‐Ad vs. Ad)	1.79	1.00–3.18	0.0486	2.37	1.29–4.35	0.0052
Symptoms from BMs (yes vs. no)	1.36	0.82–2.24	0.2310			
Extracranial metastases (present vs. absent)	0.81	0.47–1.40	0.4570			
Primary cancer status (controlled vs. not controlled)	0.55	0.34–0.91	0.0194	0.38	0.22–0.66	0.0006
Number of BMs	1.04	0.97–1.12	0.2370			
Maximum diameter	1.07	0.86–1.32	0.5515			
Total volume of BMs	1.00	0.99–1.01	0.8415			
KPS	0.99	0.97–1.00	0.1068			
Prior chemotherapy (yes vs. no)	2.11	1.22–3.64	0.0073	3.36	1.85–6.10	<0.0001
Prior targeted therapy (yes vs. no)	0.95	0.57–1.58	0.8521			
Salvage treatment (yes vs. no)	2.03	1.22–3.39	0.0067	2.16	1.26–3.70	0.0052

Abbreviations: Ad, adenocarcinoma; BMs, brain metastases; CI, confidence interval; HR, hazard ratio; KPS, Karnofsky Performance Scale; y, years.

### SRS‐related adverse events

Eight patients developed radionecrosis after SRS, of which four were symptomatic, and four were asymptomatic. The median time to the diagnosis of radionecrosis was 11 (range 3–28) months. Neurological deficits associated with radionecrosis included seizures, motor deficits, cognitive deficits, and speech deficits.

## DISCUSSION

For decades, SRS has been used to treat BMs to deliver therapeutic doses of tumor irradiation while minimizing damage to adjacent normal tissue. Current evidence‐based guidelines support SRS without concurrent WBRT for patients with up to four BMs.^4^, ^15^ However, debate continues about how many tumors can be treated with SRS alone. Two multi‐institutional studies have investigated the concerns. In a retrospective review of 243 patients treated with SRS, patients with <5 BMs did not show significantly improved OS in a multivariable model compared with patients with ≥5 BMs.^13^ In addition, a prospective observational study conducted by the Japanese Leksell Gamma Knife Society evaluated 1194 patients. The results suggested that SRS without WBRT in patients with five to ten BMs was not inferior to that in patients with two to four BMs.^5^

Most of the published literature included highly heterogeneous patients with discordant primary cancer types. Because various primary malignancies exhibit different radiosensitivity and clinical responsiveness, our study focused only on NSCLC. The median OS in this study was 17.7 months, and the median IPFS was 9.2 months, similar to the previously reported results.^16^, ^17^, ^18^ There were no significant differences in OS and IPFS between the <5 BMs group and ≥5 BMs group. Furthermore, the univariate and multivariate analyses showed tumor number was not a significant predictor for OS and IPFS. Our findings suggested that SRS alone as the initial treatment for NSCLC patients with more than four BMs was non‐inferior to SRS for those with 1–4 BMs in terms of OS and IPFS.

Previous studies have shown that the independent prognostic factors for survival in patients with BMs treated with SRS included histology, gender, age, KPS, neurological symptoms, system disease control, chemotherapy, targeted therapy, and salvage treatment.^13^, ^14^, ^16^, ^17^ Meanwhile, our results indicated that histology, primary cancer status, KPS, and salvage treatment could independently predict patients' OS. In brief, the OS of patients with adenocarcinoma was significantly longer than that of patients with other NSCLCs. Patients with higher KPS showed higher survival rates. Previously published literature supported these results.^14^, ^16^

We found that the number of BMs correlated with extracranial metastases and primary cancer status, resulting in the differences in patient characteristics (extracranial metastases and primary cancer status) between the <5 BMs and ≥5 BMs groups, possibly because of the metastasis to the brain and other organs promoted by uncontrolled primary cancer. Knoll et al.^13^ also found close correlations between the number of intracranial metastases, systemic disease status, and extracranial metastatic burden. Further, they demonstrated system disease status instead of the number of intracranial metastases to be an independent predictor.^13^ Likewise, we found that primary cancer status was an independent predictor of survival.

In our study, prior chemotherapy and targeted therapy had no significant effect on survival after SRS. The development of novel chemotherapeutic agents for lung cancer improved survival. In particular, targeted agents show superior pharmacokinetics in penetrating through the blood–brain barrier, which would reduce the incidence of intracranial progression and lead to better survival. Our sample size may have been too small to highlight a difference in survival based on prior chemotherapy and targeted therapy. In addition, we did not include subsequent chemotherapy and targeted therapy in the analysis of prognostic factors because of a lack of relevant data.

There is now a shift to using repeat SRS instead of WBRT to treat distant recurrent BMs after initial SRS,^19^ as in our study; most of the salvage treatment for recurrent BMs was repeat SRS. Because of advances in systemic chemotherapy and targeted therapy, NSCLC patients can achieve more prolonged survival. Their prolonged survival, however, increases the risk of intracranial progression. SRS is an excellent option for salvage treatment because it can be repeated if new lesions appear, whereas WBRT can only be performed once. Using SRS for BMs and reserving WBRT for leptomeningeal dissemination after SRS provides a significant clinical advantage. Benjamin et al.^20^ also believed that multiple SRS courses were feasible and safe for selected patients with a large number of cumulative BMs.

This study has several limitations. First, it was a retrospective study with a small sample size. Second, detailed adverse events were not described because most patients were followed up by telephone and the relevant data were incomplete. Third, data on subsequent chemotherapy and targeted therapy were missing because most patients received systemic treatment in outside institutions.

In conclusion, our study demonstrated that SRS alone as the initial treatment for NSCLC patients with more than four BMs was non‐inferior to SRS for those with one to four BMs in terms of OS and IPFS. Further study is required to explore SRS toxicity for patients with ≥5 BMs.

## CONFLICT OF INTEREST

No authors report any conflict of interest.
